# Cardiovascular responses of adult male Sprague–Dawley rats following acute organophosphate intoxication and post-exposure treatment with midazolam with or without allopregnanolone

**DOI:** 10.1007/s00204-023-03679-x

**Published:** 2024-02-02

**Authors:** Shiyue Pan, Donald A. Bruun, Pamela J. Lein, Chao-Yin Chen

**Affiliations:** 1 Department of Pharmacology, Davis, School of Medicine, University of California, Davis, CA USA; 2 Department of Molecular Biosciences, Davis, School of Veterinary Medicine, University of California, Davis, CA USA; 3 Davis, School of Medicine, MIND Institute, University of California, Sacramento, CA USA

**Keywords:** Organophosphate, Autonomic function, Heart rate variability, Baroreflex sensitivity, Arrhythmia

## Abstract

Recent experimental evidence suggests combined treatment with midazolam and allopregnanolone is more effective than midazolam alone in terminating seizures triggered by acute organophosphate (OP) intoxication. However, there are concerns that combined midazolam and allopregnanolone increases risk of adverse cardiovascular events. To address this, we used telemetry devices to record cardiovascular responses in adult male Sprague–Dawley rats acutely intoxicated with diisopropylfluorophosphate (DFP). Animals were administered DFP (4 mg/kg, sc), followed immediately by atropine (2 mg/kg, i.m.) and 2-PAM (25 mg/kg, i.m.). At 40 min post-exposure, a subset of animals received midazolam (0.65 mg/kg, im); at 50 min, these rats received a second dose of midazolam or allopregnanolone (12 mg/kg, im). DFP significantly increased blood pressure by ~ 80 mmHg and pulse pressure by ~ 34 mmHg that peaked within 12 min. DFP also increased core temperature by ~ 3.5 °C and heart rate by ~ 250 bpm that peaked at ~ 2 h. Heart rate variability (HRV), an index of autonomic function, was reduced by ~ 80%. All acute (within 15 min of exposure) and two-thirds of delayed (hours after exposure) mortalities were associated with non-ventricular cardiac events within 10 min of cardiovascular collapse, suggesting that non-ventricular events should be closely monitored in OP-poisoned patients. Compared to rats that survived DFP intoxication without treatment, midazolam significantly improved recovery of cardiovascular parameters and HRV, an effect enhanced by allopregnanolone. These data demonstrate that midazolam improved recovery of cardiovascular and autonomic function and that the combination of midazolam and allopregnanolone may be a better therapeutic strategy than midazolam alone.

## Introduction

Organophosphate cholinesterase inhibitors (OP) include pesticides widely used in agriculture as well as nerve agents used as chemical threat agents. Acute poisoning with organophosphorus cholinesterase inhibitors (OPs), such as OP nerve agents and pesticides, due to occupational, intentional, and terrorism-related exposures is the cause of hundreds of thousands of deaths each year (Brooks et al. 2018; Mew et al. 2017). Acute OP intoxication leads to accumulation of acetylcholine and hyperstimulation of the cholinergic pathway both in the peripheral and the central nervous system (CNS), resulting in cholinergic crisis (Hulse et al. 2014). Without intervention, the primary cause of death from OP-induced cholinergic crisis is respiratory failure (Bugay et al. 2022; Dube et al. 1993; Gaspari and Paydarfar 2007; Hulse et al. 2014). Cardiovascular collapse from direct effects of OP on the cardiovascular system also contribute to fatal outcomes (McFarland and Lacy 1968; Munidasa et al. 2004).

Acute OP intoxication has been shown to produce variable cardiovascular effects, including hypertension, hypotension, tachycardia, and bradycardia (Dube et al. 1993; Gordon and Padnos 2000). Factors such as route of exposure (rate of absorption), treatment intervention, exposure dose, and time of measurement contribute to the inconsistency in reported human cardiovascular effects while the use of anesthetized/restraint procedures for cardiovascular measurements in animal models introduces additional confounding variables (Dube et al. 1993; Gordon and Padnos 2000). Thus, one goal of this study was to longitudinally monitor cardiovascular responses to acute intoxication with the OP, diisopropylfluorophosphate (DFP), in conscious, freely moving rats using continuous telemetry recordings.

Acute OP intoxication can also trigger seizures that rapidly progress to life-threatening status epilepticus (de Araujo et al. 2012). The benzodiazepine, midazolam, is the standard of care for treating OP-induced seizures (Newmark 2019). However, benzodiazepines do not completely prevent OP-induced progressive neurodegeneration and spontaneous recurrent seizures (Dhir et al. 2020). Using allopregnanolone as an adjunct to midazolam has been shown to reduce the mean seizure scores more than midazolam alone in a rat model that recapitulates the acute and long-term neurological effects of acute OP intoxication in human (Dhir et al. 2020). However, we have no information on the cardiovascular and autonomic effects of these anti-seizure treatments, Moreover, since both midazolam and allopregnanolone are positive allosteric modulators of γ-aminobutyric acid-A (GABA_A_) receptors, there are concerns that use of these drugs may augment adverse cardiovascular effects associated with acute OP intoxication. The second goal of this study, therefore, was to investigate effects of midazolam, with and without co-administration of allopregnanolone, on cardiovascular and autonomic function in rats acutely intoxicated with DFP.

## Material and methods

### Animals

All animal protocols were approved by the Institutional Animal Care and Use Committee at the University of California, Davis (protocol number 20165 approved on Oct 11, 2017 and protocol number 21954 approved on Oct 11, 2020). All animal studies therefore were performed in accordance with the ethical standards laid down in the 1964 Declaration of Helsinki and its later amendments in accordance with the National Institutes of Health guide for the care and use of laboratory animals (NIH Publications No. 8023, revised 1978) and in compliance with the ARRIVE guidelines. Adult male Sprague Dawley rats (6-8 weeks old, Charles River Laboratories, Hollister, CA) were housed individually in standard plastic shoebox cages in AAALAC accredited facilities with a 12-h light/ 12-h dark cycle and ad libitum access to regular rodent chow and water. The housing temperature was 22 ± 2 °C (mean ± SD), 40–50% humidity.

### Surgical implantation of telemetry devices

All surgeries were performed under sterile conditions. Rats were anesthetized with isoflurane (1.5–5% in 100% oxygen). The criteria for adequacy of anesthesia included: (1) no whisker movement, (2) no eye blink reflex, (3) lack of paw-pinch withdraw, and (4) no irregular or sudden changes in breathing frequency. Rats were placed on a servo-controlled water blanket and body temperature was maintained at 37 ± 1 °C. The abdominal cavity was opened through a midline incision, and the abdominal aorta was isolated below the renal artery. A blood pressure (BP) + electrocardiogram (ECG) telemetry device (HD-S11, Data Sciences International, St. Paul, MN) was implanted in the peritoneal cavity. The BP catheter was inserted into the abdominal aorta and secured with a drop of Vetbond surgical glue and a nitrocellulose patch. The biopotential leads were tunneled subcutaneously. The negative lead was sutured to the upper right pectoris muscle near the shoulder, and the positive lead was sutured to the left later side of the xiphoid process for recording ECG at lead II configuration. The transmitter body was sutured to the inside of the abdominal muscle. Rats were given Meloxicam, (2 mg/kg, sc) as preemptive analgesia and daily for two post-days for pain management. Rats were allowed to recover from the surgery for two weeks. During the recovery period, rats were checked daily for signs of pain such as excessive grooming and inactivity.

### DFP intoxication

DFP (Sigma Chemical Company, St Louis, MO, USA) was aliquoted and stored at − 80 °C, storage conditions previously shown to maintain DFP stability for one year (Heiss et al. 2016). DFP purity, which was monitored in-house using 1H-13C, 19F and 31P-NMR methods, as previously described (Gao et al. 2016), was approximately 90 ± 7%. During experiments, DFP was diluted with ice-cold sterile phosphate-buffered saline (PBS, 3.6 mM Na2HPO4, 1.4 mM NaH2PO4, 150 mM NaCl; pH 7.2) within five min of administration. Unanesthetized rats were injected with 300 µl DFP (4 mg/kg, sc) subcutaneously in the subscapular region to induce status epilepticus (Flannery et al. 2016; Guignet et al. 2020). To decrease mortality from cholinergic crises (Bruun et al. 2019), animals were treated within 1 min after DFP injection with 2 mg/kg, im atropine sulfate (Sigma, lot #BCBM6966V, > 97% pure) and 25 mg/kg, im pralidoxime (2-PAM; Sigma, lot #MKCG3184, > 99% pure). Both drugs were diluted in sterile isotonic saline (0.9% NaCl). Vehicle (VEH) control animals were injected sc with 300 µl sterile PBS in place of DFP but were similarly treated with atropine and 2- PAM. A random number generator was used to randomly assign animals to experimental groups. Behavioral seizures were monitored and recorded every 5 min for the first 2 h post-exposure and every 20 min for 2–4 h post-exposure using a modified Racine scale (Deshpande et al. 2016; Pouliot et al. 2016). Previous studies have demonstrated that a seizure score ≥ 3.0 is commensurate with electroencephalographic seizure activity (Deshpande et al. 2016) and an average behavioral seizure score ≥ 2.5 is indicative of status epilepticus (Deshpande et al. 2016; Pouliot et al. 2016). All DFP-intoxicated rats used in this study had an average behavioral seizure score of 2.5 or higher during the first hour post-DFP. At the end of the 4-h seizure monitoring period, rats were administered 5% dextrose in saline and placed in their home cage and provided with moistened chow.

### Midazolam/allopregnanolone treatment paradigm

In a separate group of animals, rats were implanted with a telemetry device two weeks before DFP exposure. Atropine sulfate (2 mg/kg, i.m.) and 2-PAM (25 mg/kg, i.m.) were given within 1 min after DFP (s.c.) injection. Rats that survived to the 40 min mark received midazolam (0.65 mg/kg, i.p.) followed 10 min later by either a second dose of midazolam (0.65 mg/kg, i.p.) or allopregnanolone (12 mg/kg, i.p.). A 40 min was selected because it represents the average time a person can reasonably obtain medical treatments after acute OP intoxication (Wu et al. 2018); and this dosing paradigm is consistent with the CHEMM guidelines for treating OP-induced seizures, which stipulate a 10-min delay between the first and second dose of midazolam.

### Data acquisition and analysis

Continuous ECG signals were recorded at 4-kHz, BP signals were recorded at 500-Hz, and core temperature was recorded every minute with Ponemah software (Data Sciences International, St Paul, MN). The DSI telemetry system has an algorithm that estimates the animal’s activity based on the strength of the signal transmitted to the receiver, taking into consideration both the orientation of the animal relative to the receiver and the distance from the animal to the receiver antennas. The activity level was expressed as counts/min. In the acute cardiovascular response to DFP intoxication with no treatment experimental group, BP, ECG, and core temperature were recorded starting 2 h before DFP or vehicle injections and up to 24 h post DFP/ vehicle injections. Rats with DFP injections were divided into three groups based on their survival time after DFP exposure: survived less than 15 min (group I), survived greater than 15 min but less than 17 h (group II) and survived more than 17 h (group III). The 17-h criterion was chosen because this was the earliest time in which the recording was terminated for the acute intoxication assessment.

**Fig. 1:**
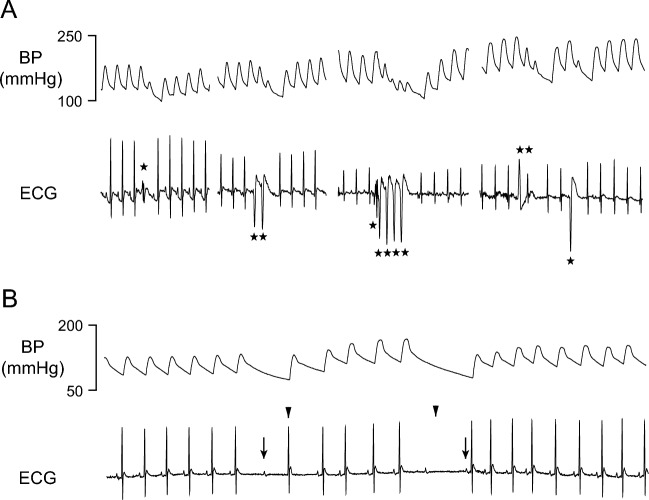
Examples of ventricular **A** and non-ventricular **B** cardiac events. Asterisks: pre-mature ventricular complexes. Triangles: AV blocks. Arrows: sinus bradycardia

Systolic BP (SBP), diastolic BP (DBP), mean BP (MBP), pulse pressure (PP), and heart rate (HR) were determined offline using the Ponemah software (Data Sciences International). To quantitate arrhythmia, ECG waveforms were visually inspected 30 min before DFP injections (all groups), the first 30 min after DFP injections (all groups), and during the last hour of survival (group II only). Two types of cardiac events were classified (Fig. 1): ventricular (pre-matured ventricular complexes) and non-ventricular (supra-ventricular) events. Ventricular events appeared as a single, double, or multiple ectopic pre-matured ventricular complexes either from presumed same focus or multiple foci (Fig. 1A). Non-ventricular events included sinus bradycardia and atrial-ventricular blocks of various degrees (Fig. 1B).

In the anti-seizure treatment experiments, continuous BP, ECG and core temperature were recorded starting 2 d before DFP injection to determine baseline physiologic levels, and up to12 d post-DFP injection. In addition to BP and HR readouts, standard time domain heart rate variability (HRV) was used to assess autonomic function. For HRV analysis, R waves from ECG waveforms were marked with Ponemah software (Data Sciences International, St Paul, MN, USA). Key to accurate HRV analysis is that only normal-to-normal RR intervals (RRI) are included in the analysis (Malik et al. 1996). After the initial R wave detection with the Ponemah software, two additional criteria were used to exclude abnormal RR intervals (RRI) using Data Insights software (Data Sciences International): (1) any RRI longer than 600 ms (HR < 100 bpm) were excluded (from either non-physiological bradycardia, AV block or the program failed to identify an R wave); and (2) any RRI that differed by ≥ 20% from either adjacent RRI were excluded (mostly from pre-matured ventricular complexes or first degree AV block) (Karey et al. 2019). This 20% exclusion approach has been shown to have the best sensitivity in correctly including normal RRI and specificity in eliminating abnormal RRI and, thus, providing reliable estimates of time domain HRV measures from long-term continuous recordings in rodents (Karey et al. 2019). Standard short-term HRV (RMSSD, root mean square of successive difference) and overall HRV (SDNN, standard deviation of normal-to-normal RR interval) were calculated.

### Statistical analysis

Data are expressed as mean ± SE unless indicated otherwise. GraphPad Prism (GraphPad Software, Inc.) was used for all statistical analyses. Data from 30 min before vehicle or DFP injections were averaged as baseline readings. Maximum response during the first 15 min of post-injection period were used as peak responses. Injection-induced changes on cardiovascular parameters were compared with a paired t test (baseline vs. peak response). DFP- and vehicle-induced changes on cardiovascular parameters were calculated by subtracting baseline from the peak response and analyzed with a one-way ANOVA. Tukey’s post-hoc tests were performed when appropriate. DFP-induced ventricular events were analyzed with Mixed-effects model.

To assess effects of anti-seizure treatments on acute DFP responses, we obtained 10 min averages for the first 2 h after DFP injections and hourly averages thereafter (for up to 8 h post-DFP). Additionally, 12 h averages were calculated from 36 h before DFP injection to 11 d post-DFP. These data were analyzed with a two-way repeated measure ANOVA (time x drug treatment) followed by Fisher’s LSD tests when appropriate. *p* < 0.05 was considered statistically significant.

## Results

Consistent with the 10-year history of working with the rat model of acute DFP intoxication in our laboratory, we observed a 66.7% survival rate in the cohort of all DFP-intoxicated animals used in this study.

We recorded cardiovascular parameters from 11 rats that died within the first 15 min of DFP intoxication (Group I, averaging 9.1 ± 0.5 min), nine rats that died before termination of the recording (Group II, ranging from 1.7 to 14.6 h post-DFP exposure), and four rats that survived to the end of recordings (Group III, > 17 h post-DFP). In addition, eight rats were injected with vehicle as the injection procedure control.

**Fig. 2:**
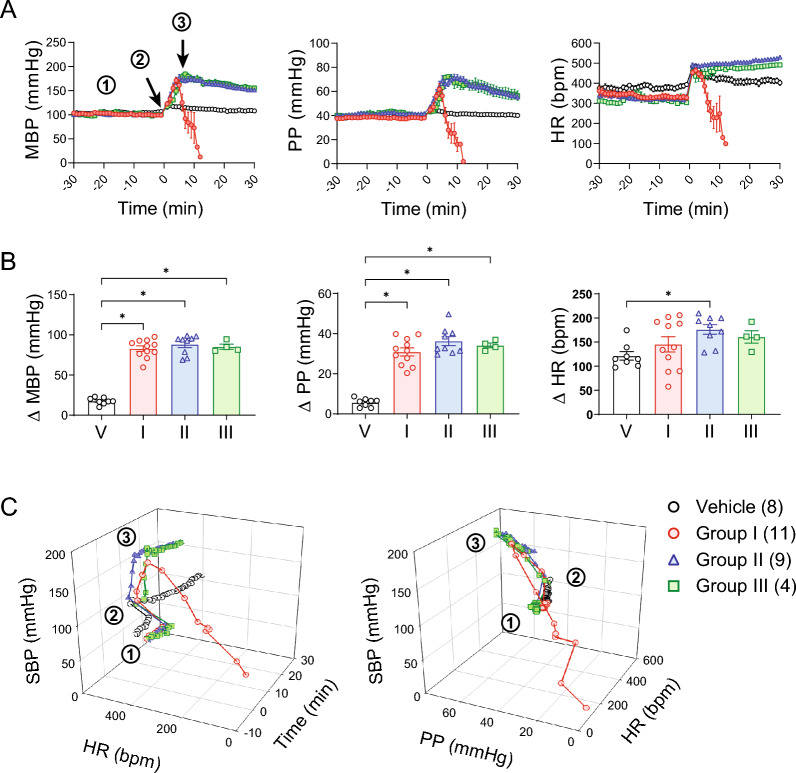
Group data of cardiovascular responses to DFP intoxication. **A** Cardiovascular responses over time. The pressor response peaked at around 5–12 min post DFP injection. Group I had abrupt drops in MBP and HR just before or around the peak pressor response. Groups II and III had a slow, continuous increase in HR over the first 30 min post-DFP. **B** Peak increases in MBP (one way ANOVA, *p* < 0.0001), PP (one way ANOVA, *p* < 0.0001), and HR (one way ANOVA, *p* = 0.0435) in the first 15 min post-DFP. **C** 3D plots of SBP and HR over time (left) and SBP, PP, and HR (right). *MBP* mean blood pressure, *SBP* systolic blood pressure, *PP* pulse pressure, *HR* heart rate. *p < 0.05, Tukey’s post-hoc test

In conscious rats, administration of DFP (4 mg/kg, s.c.), followed by blocking peripheral cholinergic toxicity with atropine and 2-PAM, increased BP, PP and HR (Fig. 2A). In the groups that survived longer than 15 min (Groups II and III), the pressor response peaked at around 5–12 min. Rats that died within 15 min (Group I) had abrupt drops in BP and HR just before or around the peak pressor responses seen in groups II and III. Vehicle injections induced a small transient but significant increase in MAP (104 ± 2 vs. 122 ± 2 mmHg, baseline vs. peak respectively, *p* < 0.05, paired *t*-test), pulse pressure (39 ± 1 vs. 45 ± 1 mmHg, baseline vs. peak respectively, *p* < 0.05, paired *t*-test), and HR (378 ± 11 vs. 496 ± 10, baseline vs. peak respectively, *p* < 0.05, paired *t*-test). DFP-induced pressor responses (~ 85 mmHg for MBP and ~ 34 mmHg for PP) were significantly greater than those of vehicle control (Fig. 2B). The pressor response did not differ among the three DFP groups. The immediate increase in HR after DFP injection was likely a response to the handling/ injection procedure as the vehicle group had a similar initial increase in HR (Fig. 2B). For groups II and III, HR showed a slow, continuous increase over the first 30 min post-injection period, while the HR in the vehicle group gradually returned to baseline (Fig. 2A). When looking at minute-averages of BP and HR together over time (Fig. 2C), both parameters dropped simultaneously in rats from group I (Fig. 2C, left). Furthermore, these decreases were accompanied with a concurrent decrease in PP (Fig. 2C, right).

**Fig. 3:**
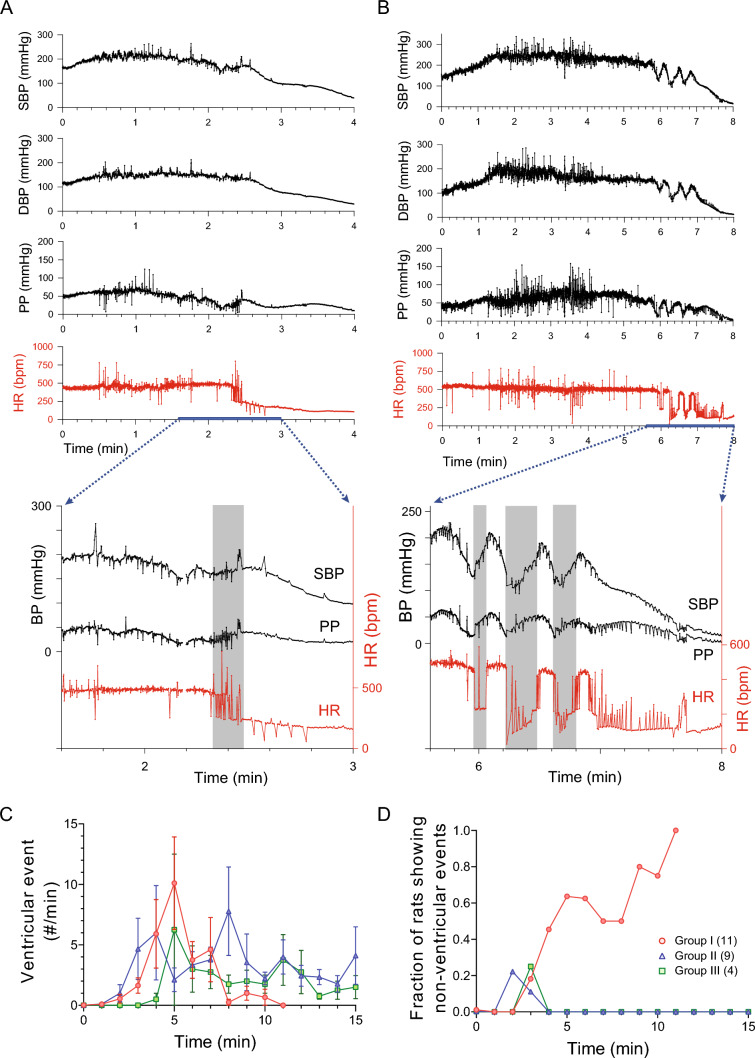
DFP-induced cardiac arrhythmia. **A** An example of systolic blood pressure (SBP), diastolic blood pressure (DBP), pulse pressure (PP), and heart rate (HR) from a rat in Group I showing significant bradycardia minutes before death. **B** An example of SBP, DBP, PP, and HR from a rat in Group I showing repeated bradycardic episodes before death. **C** Ventricular event counts after DFP injection. **D** Fraction of rats displayed non-ventricular event after DFP injection

Two representative plots of post-DFP beat-to-beat SBP and HR from group I are shown in Fig. 3. After the initial rise in BP, all rats showed an increased swing in beat-to-beat SBP and HR, originating from ventricular events (pre-matured ventricular complexes). All rats in group I showed significant bradycardia minutes before they died, either from a single episode (Fig. 3A) or in a re-current pattern (Fig. 3B). Figure 3B shows a typical re-current pattern that started with a drop in PP and BP, followed by a significant sinus bradycardia. The subsequent increase in BP and PP during the bradycardia period was likely mediated by the Starling’s mechanism from an increased pre-load associated with the slowing of HR. These data suggest that a significant sinus bradycardia initiated the cardiovascular collapse that resulted in death.

Rats that survived longer than 15 min (groups II and III) also had an increased swing in beat-to-beat SBP and HR (also originating from pre-matured ventricular complexes) after the initial rise in BP. Figure 3C shows the number of ventricular events (minute averages) of the first 15 min after DFP injections. There was no difference among the three groups (*p* > 0.05, Mixed-effects model). These data suggest that the occurrence of ventricular events is not a good predictor for survival. Strikingly, all rats in group I had non-ventricular cardiac events minutes prior to death while only a couple of isolated such events were observed in groups II and III (Fig. 3D). These data suggest that autonomic dysfunction may contribute to the DFP-induced acute cardiovascular collapse.

**Fig. 4:**
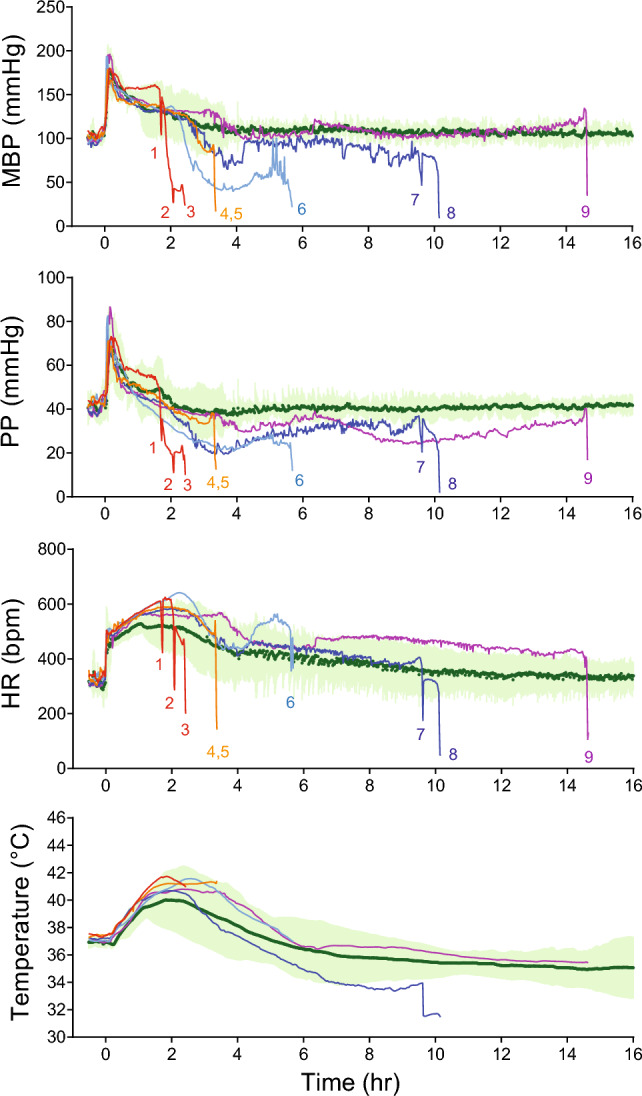
Cardiovascular and temperature responses to DFP intoxication. Group data from rats in group III (in green) are shown as mean ± 95% CI. Rats in group II are numbered according to their survival time. Red, rats #1–3; orange, rats #4–5; teal, rat #6; blue, rats #7–8; purple, rat #9

The time course of DFP-induced changes in cardiovascular parameters and core temperature in survivors (group III) are shown in Fig. 4 (in green, expressed as mean ± 95% CI). After the initial rise, BP and PP returned to baseline level over the next few hours. For HR, after the initial rapid post-injection increase, HR continued with a slower increase over the next two hours. Core temperature had a gradual increase and peaked ~ 2 h post injection. Group II rats had similar temperature response to DFP intoxication as those survivors (group III).

A pattern of cardiovascular collapse similar to that seen in group I was observed in group II (Fig. 4). For clarity, rats in group II were numbered (# 1–9) in the sequence of their survival time. A closer inspection of cardiovascular parameters in each of the nine rats from group II showed that one rat had simultaneous decrease in MBP, PP, and HR (#1). Five rats (# 2–6) had significant decrease in MBP and PP with maintained HR before a significant rapid drop in HR that followed by death. The remaining three rats (# 7–9) showed recovery from the decrease in MBP and PP before the cardiovascular collapse. These data raised the possibility that reduced cardiac contractility may contribute to the cardiovascular collapse at later times post-exposure.

**Fig. 5:**
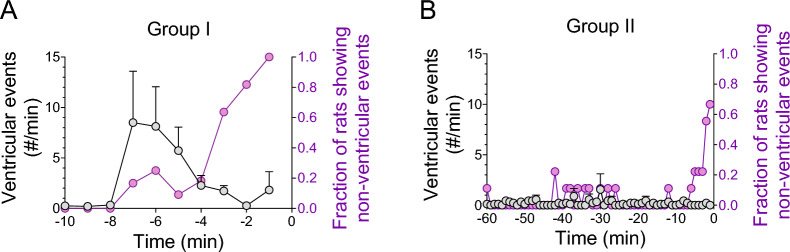
Group data of averaged ventricular events (grey) and fraction of rats showing non-ventricular events (purple) in group I (**A**) and group II (**B**). Time zero is the time of death

Minute averages of ventricular events and occurrence of non-ventricular events were plotted based on the time of the animal’s death (time zero), regardless of their survival time or BP level (Fig. 5). Rats in group I showed a drop in the ventricular events minutes before death, while the probability of developing non-ventricular events increased (Fig. 5A). All animals in group I had non-ventricular events minutes before death. For group II, there was no significant ventricular event counts an hour before death (Fig. 5B). However, six out of the nine rats displayed non-ventricular events within 10 min of death. These data suggest that the occurrence of non-ventricular events may be a better predictor for cardiovascular collapse than ventricular events.

**Fig. 6:**
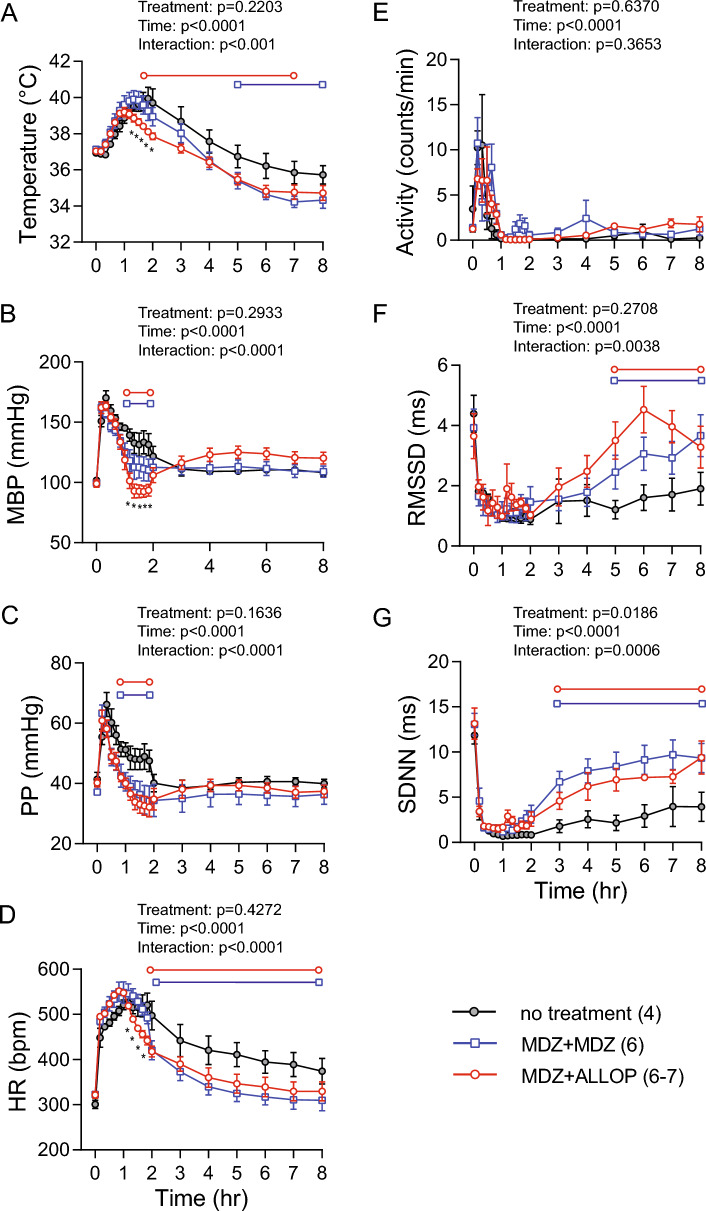
Group data of first 8 h of antiseizure treatments. Rats treated with midazolam + allopregnanolone had reduced hyperthermia response compared to the midazolam alone and no treatment groups (**A**). Both treatment groups significantly facilitated the recovery from the initial increases in MBP (**B**), PP (**C**), and HR (**D**). Compared to midazolam alone group, the allopregnanolone group had significantly lower MBP and HR between hour 1 and 2 post-DFP. There was no difference in activity level among the three groups (**E**). Both treatment paradigms significantly improved the recovery of short-term (**F**) and overall (**G**) HRV. *MBP* mean blood pressure, *HR* heart rate, *PP* pulse pressure, *RMSSD* root mean square of successive difference, *SDNN* standard deviation of normal-to-normal RR intervals. Numbers in parentheses indicate number of rats. Data were analyzed with a two-way repeated measure ANOVA, followed by Fisher’s LSD tests when appropriate. Horizontal bars indicate data points that were significantly different from the no treatment group (red line with circles, midazolam + allopregnanolone; blue line with squares, midazolam alone). *p < 0.05 midazolam + allopregnanolone (MDZ + ALLOP) vs. midazolam alone (MDZ)

Compared to treatment with midazolam alone, the addition of allopregnanolone significantly dampened the acute hyperthermia response (Fig. 6A). Midazolam treatment, with or without allopregnanolone, significantly improved the recovery from the initial increases in BP, PP, and HR (Fig. 6B–D). Addition of allopregnanolone improved recovery, as evidenced by significantly lower MBP and HR at 1–2 h post-DFP in the midazolam + allopregnanolone group compared to the midazolam alone group (Fig. 6B, D). There was no difference in PP (Fig. 6C) or activity level (Fig. 6E) between the two treatment groups. Both short-term (RMSSD) and overall HRV (SDNN) were significantly suppressed during the first 2 h after DFP injection (Fig. 6F, G), regardless of whether the animal received anti-seizure treatments. Both anti-seizure treatments significantly improved the recovery of these HRV parameters, suggesting an improved autonomic function compared to the DFP animals that did not receive anti-seizure medications.

**Fig. 7:**
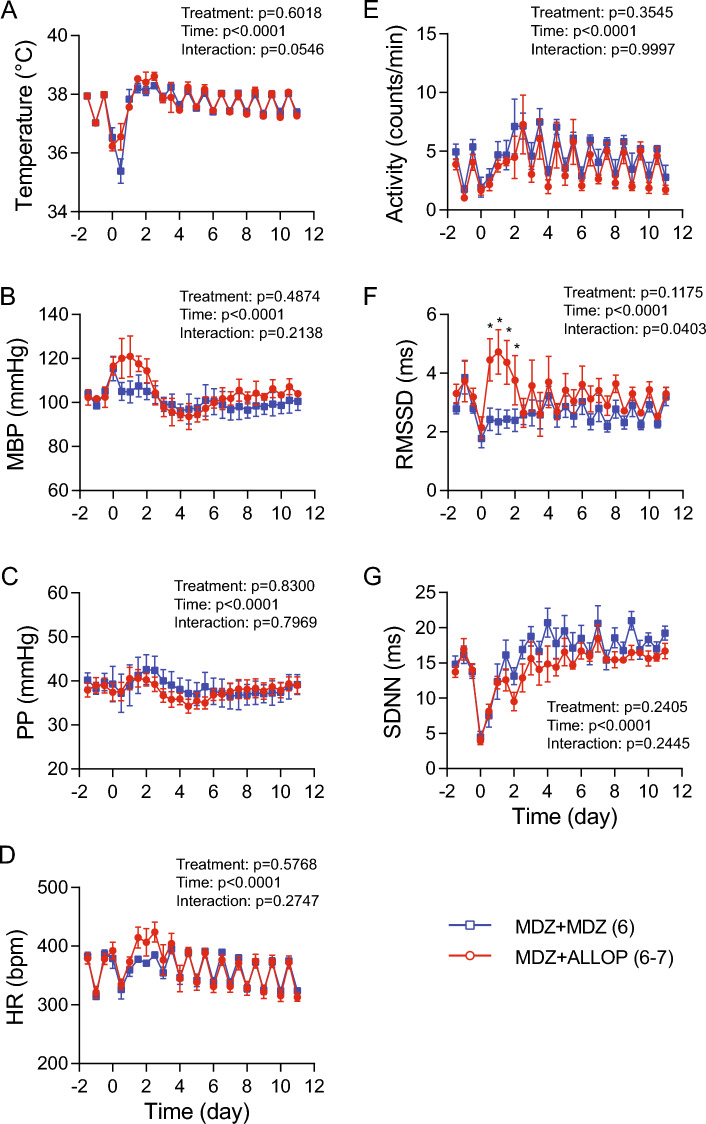
Group data over 11 d after administration of anti-seizure treatments. There was no difference between the two treatment groups in core temperature (**A**), activity level (**B**), MBP (**C**), HR (**D**), PP (**E**), BRS (**F**), and SDNN (**H**). The allopregnanolone-treated group had significantly higher short-term HRV the first 2 d post-treatment (**G**). *MBP* mean blood pressure, *HR* heart rate, *PP* pulse pressure, *RMSSD* root mean square of successive difference, *SDNN* standard deviation of normal-to-normal RR intervals. Numbers in parentheses indicate number of rats. Data were analyzed with a two-way repeated measure ANOVA, followed by Fisher’s LSD tests when appropriate. *p < 0.05 midazolam + allopregnanolone (MDZ + ALLOP) vs. midazolam alone (MDZ)

Figure 7 shows 12 h averages over 11 d post-treatment. There was a trend, but not statistical significance, for a better maintained core temperature on day 1 in the allopregnanolone-treated group compared to the midazolam alone group (Fig. 7A). No differences between the two treatment groups were observed in activity, MBP, PP, HR, and overall HRV (SDNN). However, the allopregnanolone-treated group had significantly higher short-term HRV the first 2 d post-treatment (Fig. 7F), suggesting that the addition of allopregnanolone in the treatment may better maintain autonomic function.

## Discussion

There are four major findings in this study. *First*, our data showed that acute DFP intoxication with atropine and 2-PAM on board induced a significant hypertensive response that gradually returned to baseline over 2–3 h post-DFP injection, while HR and core temperature gradually increased over the first 2 h post DFP injection. *Second*, all rats that expired within 15 min of DFP injection (group I) developed significant non-ventricular events (sinus bradycardia and AV blocks) within minutes of death. For those rats that survived past 15 min but expired hours after DFP intoxication (group II), two-thirds also developed non-ventricular events within 10 min of death. These data suggest that DFP-induced autonomic dysfunction may contribute to acute cardiovascular collapse and that non-ventricular events should be closely monitored in patients with acute OP intoxication. *Third*, midazolam treatment, with or without allopregnanolone, significantly improved the recovery of cardiovascular parameters and autonomic function. These data suggest that anti-seizure treatment also promotes cardiovascular recovery, potentially by restoring autonomic function. *Fourth*, compared to the midazolam alone group, the midazolam with allopregnanolone group had better cardiovascular improvement for the first 2 h and better autonomic function in the first 2 d post-DFP exposure. These data suggest that adding allopregnanolone to the treatment may have added cardiovascular benefits.

Our results showing an initial hypertensive response with a more delayed hypotension effect are consistent with a number of studies, including studies of rats with DFP intoxication (Yen et al. 2001), rabbits with paraoxon exposure (Kullmann and Uerdingen 1978), and hens with tabun exposure (Worek et al. 1995). Smith and colleagues showed that administration of a muscarinic receptor agonist decreased BP but a hypertensive response was observed when co-administered with a peripheral muscarinic receptor antagonist, suggesting that activation of central muscarinic receptors increased BP (Smith et al. 2001). Varagic showed that administration of hexamethonium abolished eserine-induced increase in BP (Varagic 1955). Similarly, Dirnhuber and Cullumbine showed that sarin-induced hypertension was abolished by transecting the spinal cord between C1 and C2, suggesting that the hypertensive response was mediated by central activation of the sympathetic nervous system (Dirnhuber and Cullumbine 1955). These data suggest that the initial hypertensive response in our study is likely due to central muscarinic receptor-mediated sympathoexcitation.

OPs have been shown to cause cardiac injury (Singer et al. 1987) that contribute to death following OP-induced cholinergic crisis (Aghabiklooei et al. 2013; Anand et al. 2009). In isolated rabbit heart, low doses of DFP induced a transient decrease in contraction force without significant changes in the ECG waveform (Knox et al. 1949; Quilliam and Strong 1947). Wolthuis and Meeter showed that DFP at 64 times the LD_50_ dose induced heart failure and subsequent death in anesthetized, atropinized, and ventilated rats (Wolthuis and Meeter 1968). They further showed that, in isolated hearts, DFP decreased contractility (to below 40% of the control level), HR, and ECG amplitude. Importantly, atropine and oxime restored HR but not the ECG amplitude or the loss of ventricular contraction force (Wolthuis and Meeter 1968). The direct effects of DFP on the heart may explain the delayed hypotension we observed in our group II rats.

Many physiological measurements and clinical health evaluations, including APACHE (acute physiology and chronic health evaluation), APS (acute physiology score), GCS (Glasgow coma scale), PSS (poisoning severity score), plasma/urine OP concentrations, acetylcholine activity, frequency components of BP and HR, BP, and FiO2, have been assessed for predicting outcomes of OP intoxication (Churi et al. 2012; Davies et al. 2008; Eizadi-Mood et al. 2007; Munidasa et al. 2004; Shadnia et al. 2007; Tsai et al. 2007; Yen et al. 2000). While most of these physiological measures and clinical evaluations showed significant associations with poor clinical outcomes, large subject variability hindered the use of these measures to predict the patients’ prognosis with certainty. In humans, about 38–67% of patients with OP poisoning showed ECG abnormality, including long QTc, PVC, AV block, ventricular tachycardia, ST elevation, T inversion, and atrial fibrillation (Chuang et al. 1996; Karki et al. 2004; Laudari et al. 2014; Paul and Bhattacharyya 2012; Saadeh et al. 1997; Yurumez et al. 2009). However, a recent study by Pannu and colleagues showed that none of these hemodynamic or ECG abnormalities was associated with in-hospital mortality (Pannu et al. 2021). Taken together, these data underscore the importance of having multiple physiological indicators for predicting patients’ outcome. With continuous ECG recordings, we found that, of all rats that died from DFP intoxication, 85% of them developed non-ventricular events within 10 min of death. These data suggest that non-ventricular events may be a good additional measure for predicting clinical health outcome and the importance of closely monitoring ECG in patients with acute OP intoxication.

While atropine and 2-PAM are standard treatments for peripheral symptoms, benzodiazepines such as midazolam, a positive allosteric modulator of GABA_A_ receptors, are the current standard of care for treating OP-induced seizures and status epilepticus (Newmark 2019). Although midazolam is effective in mitigating OP-induced seizures at the onset of seizures, it is less effective when given 40 min or later (Reddy and Reddy 2015; Wu et al. 2018). Additionally, midazolam does not prevent neuronal death when given at a delayed time, despite a reduction in seizure intensity (Spampanato et al. 2019). Dhir and colleagues showed that, when administered 40 min after DFP exposure, seizures persisted following midazolam treatment (Dhir et al. 2020). Rats treated with midazolam and allopregnanolone had significantly reduced mean seizure scores and decreased neurodegeneration compared to midazolam alone treatment (Dhir et al. 2020). Similarly, here we showed that combined treatment with midazolam and allopregnanolone significantly improved cardiovascular recovery with faster recovery when allopregnanolone was added to the treatment.

The cardiovascular effects of DFP were likely mediated by both activation of central muscarinic receptors and seizure-related increases in overall network activities. The initial hypertensive response was likely due to central muscarinic receptor-mediated sympathoexcitation (Smith et al. 2001) and the delayed hypotension may be due to, in part, to the direct effects of DFP on the cardiomyocytes (Wolthuis and Meeter 1968). Capture-related increases in overall network activities can spill over to autonomic nuclei, resulting in increases in both sympathetic and parasympathetic efferent activities (Bhandare et al. 2015; Sakamoto et al. 2008). Depending on the balance between the two autonomic branches, seizures can be associated with either tachycardia or bradycardia (Naggar et al. 2022). Similarly, the antiseizure treatments may work directly on enhancing GABAergic function in the autonomic pathways and indirectly on reducing seizure-associated increase in overall network activity. The extent to which the cardiovascular effects can be attributed to direct effects of DFP vs. seizure-related effects deserves further investigation.

Cardiac arrhythmias and severe refractory hypotension are two main causes for cardiovascular-related death following acute OP intoxication (Peter et al. 2014). While decreased cardiac function can contribute to hypotension, reduced autonomic function can contribute to increased arrhythmia risk. HRV is widely used as an index of cardiac autonomic function (Malik et al. 1996). It is widely acknowledged that reduced HRV is associated with deleterious health outcomes and is an independent risk factor for cardiac events, including arrhythmias and sudden cardiac death (Billman 2011; Kleiger et al. 2005). In general, the short-term HRV (RMSSD) reflects the cardiac vagal modulation of HR while the overall HRV (SDNN) reflects changes in both sympathetic and vagal inputs (Malik et al. 1996). Our data showed that both anti-seizure treatments improved the recovery of cardiac autonomic function (Fig. 6), suggesting that midazolam and allopregnanolone may have added benefit in reducing arrhythmia risks by improving autonomic function. Furthermore, combined treatment with midazolam and allopregnanolone better maintained short-term HRV over 2 d post-exposure, suggesting that allopregnanolone as an adjunct to standard of care for treating OP-induced seizures has multiple therapeutic benefits.

One dose of atropine was used in this study. In humans, multiple doses of atropine are commonly used to combat the cholinergic symptoms. Given the half-life of atropine (2–4 h), we would expect similar cardiovascular effects being observed in humans during the acute phase (group I of this study). While the parasympathetic limb of the autonomic nervous system continues to be blocked beyond the acute phase in humans, the sympathetic limb is less affected by atropine. Although heart rate may remain elevated, BP and PP are likely to have similar course as the present study (groups II and III). Our results may be particularly valuable for the subpopulation of patients who died after they had seemly recovered from the acute intoxication as treatment stopped.

## Data Availability

Data presented in this study are available from the corresponding author upon reasonable request.
